# A mass event of paraquat poisoning via inhalation

**DOI:** 10.3389/fpubh.2023.1309708

**Published:** 2023-12-07

**Authors:** Mengdi Shi, Mei Zeng, Tianzi Jian, Guangcai Yu, Aerbusili Genjiafu, Xiangxing Zhang, Lanlan Guo, Ruikai Shang, Zhiqiang Zhou, Tongyue Zhang, Xiangdong Jian, Baotian Kan

**Affiliations:** ^1^Department of Occupational and Environmental Health, School of Public Health, Cheeloo College of Medicine, Shandong University, Jinan, Shandong, China; ^2^Department of Poisoning and Occupational Diseases, Emergency Medicine, Cheeloo College of Medicine, Qilu Hospital of Shandong University, Shandong University, Jinan, Shandong, China; ^3^Department of Hematology, Qilu Hospital of Shandong University, Cheeloo College of Medicine, Shandong University, Jinan, Shandong, China; ^4^Department of Nursing, Qilu Hospital, Cheeloo College of Medicine, Shandong University, Jinan, Shandong, China; ^5^Department of Gerontology, Qilu Hospital, Cheeloo College of Medicine, Shandong University, Jinan, Shandong, China

**Keywords:** paraquat, acute poisoning, toxicology, respiratory, case report

## Abstract

**Objective:**

In January 2023, a rare event of collective inhalation paraquat poisoning occurred in Shandong, China. To analyze the clinical characteristics of an event of respiratory tract paraquat poisoning through inhalation.

**Methods:**

Clinical data from eight patients with paraquat inhalation poisoning were retrospectively analyzed.

**Results:**

The patients were mainly exposed to paraquat via the respiratory tract. The main clinical manifestations were ocular and respiratory irritation. Lung computed tomography (CT) showed that all eight patients had varying degrees of lung injury, mainly manifesting as exudative lesions. Laboratory tests revealed arterial blood gas hypoxemia, abnormal white blood cell count, and increased neutrophil ratio. Sufficient glucocorticoid impact therapy was effective, and all eight patients survived.

**Conclusion:**

Eight patients experienced chest tightness, shortness of breath, and varying degrees of lung injury due to inhalation of paraquat through the respiratory tract. The early use of glucocorticoids and other comprehensive treatment measures, active prevention and treatment of lung infections, and protection of organ function have beneficial effects in such cases.

## 1 Introduction

Paraquat is a highly effective non-selective contact herbicide that is also highly toxic to humans and animals, and is a significant cause of death due to pesticide poisoning in China (1–3). Paraquat poisoning is typically caused by oral ingestion of the substance, but rare cases of poisoning caused by respiratory inhalation may also occur (4). On January 29, 2023, a rare cluster of paraquat inhalation poisoning incidents occurred in a city in Shandong Province. Eight patients were diagnosed with poisoning, and all patients were clinically cured after standard treatment in our department. We report their cases in detail herein.

This report has been approved by the Ethics Committee of Shandong University Qilu Hospital (Jinan, Shandong), and written informed consent for its publication was obtained from all of the patients (KYLL-2022208-008-1).

## 2 Clinical data

### 2.1 General patient information

The eight female patients were residents of the same village, aged 36–60 years, and had a mean age of (48.5 ± 9.52) years. Except for case 1, who had a history of hypertension, all of the other patients were previously healthy. All patients were married and had a history of childbearing. There were relatives between the patients, and there were elders and peers who belonged to the same large family. Their local area had recently opened a seasonal pesticide workshop, where the patients had sometimes worked as well, since November 2020. Their workloads varied in this factory according to the orders received. The average working time is about 6 h per day, the monthly work varied from 10 to 20 days, and the annual work approximately 2–3 months.

### 2.2 Poisoning event and first treatment

On January 29, 2023, the factory received an urgent order to pack a batch of herbicide particles. The eight women each transported 500 g loads of the substance from a large pile of approximately 10 tons and packed them into packaging bags. The work began at 13:00 and ended at 16:00. When working with the herbicide, the women always wore long-sleeved ordinary cloth pants and protective cotton gloves, but did not wear any protective face masks. Much more pesticide dust was produced during the packing process than usual. During such activities, many workers experience varying degrees of eye pain, tears, and other symptoms. After a short break, these symptoms often do not improve significantly. A number of patients also experience hoarseness, speech difficulties, and chest tightness symptoms. Our cases were also affected by such occurrences and visited the village clinic at 17:00 later the same day, where they were given infusions and other symptomatic treatments. These did not relieve their symptoms; therefore, at 20:00, they were admitted to the local county hospital for further treatment. Chest computed tomography (CT) scans revealed that some of the patients had lung inflammation. These serious patients were then admitted to the intensive care unit (ICU). On January 31, a toxicology test report showed that the patients’ blood and urine contained paraquat. The paraquat concentration of case 1 was 4 ng/mL in blood and 3 ng/mL in urine; for case 4, it was 6 ng/mL in blood and 7 ng/mL in urine.

On the evening of February 1, doctor in our department was contacted for an urgent consultation. The patients were diagnosed with paraquat poisoning. At 1:00 on February 2 (approximately 80 h after the exposure), the eight patients were transferred to our department for treatment.

### 2.3 Condition changes and treatment

**Figure 1:**
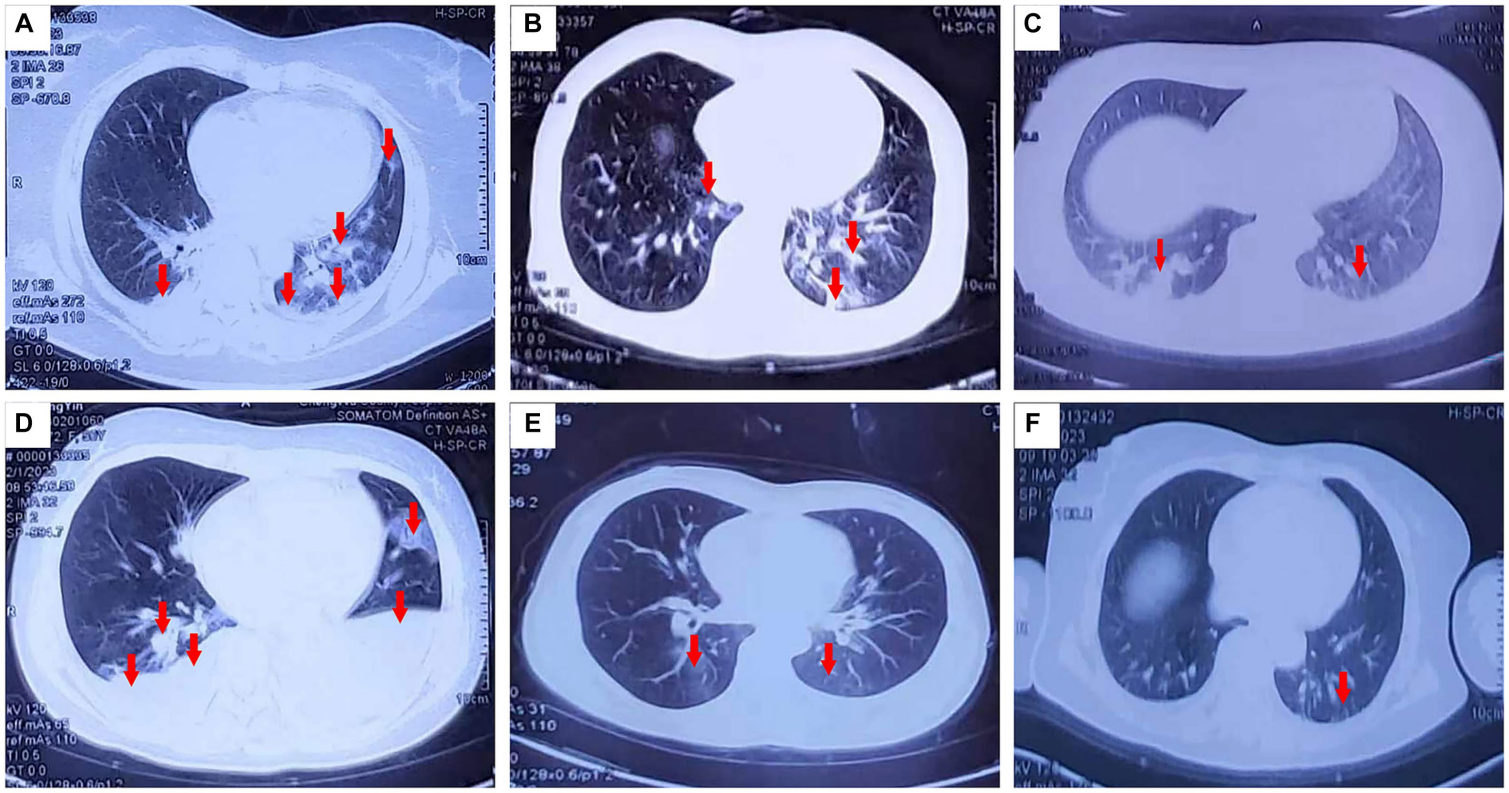
Pulmonary CT changes were observed 3 days (February 1, 2023) after exposure to paraquat powder. (**A**–**D**,**E**,**F** correspond to cases 1–4 and 7–8).

**Figure 2:**
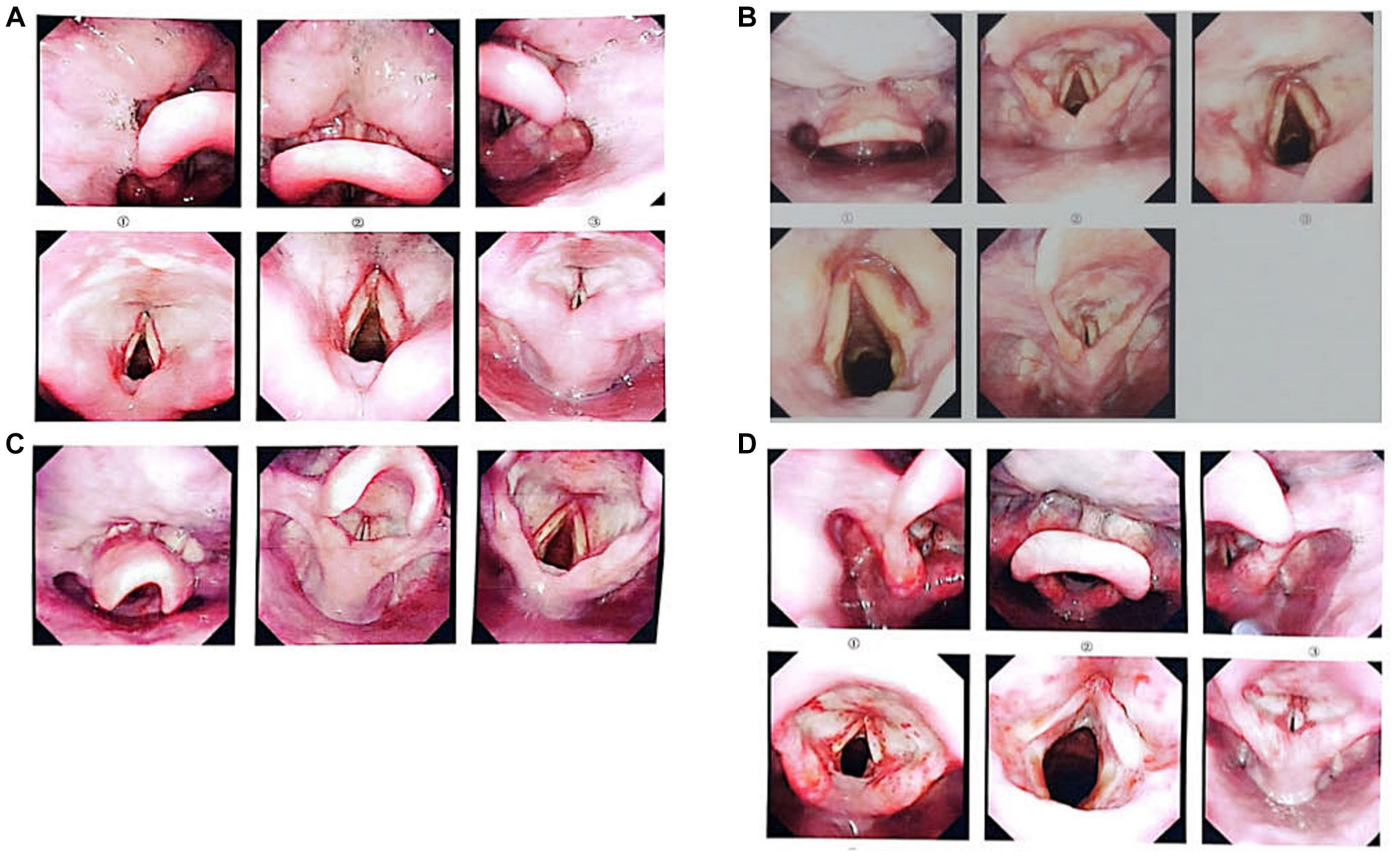
Images taken by laryngoscopy (**A–D** correspond to cases 1–4). Laryngoscopy was performed on February 7, 2023, for cases 1, 3, and 4; for Case 2, it was performed on February 8, 2023. Panel **(A)** shows that the mucous membranes of the bilateral ventricular bands, laryngeal ventricles, and both vocal cords were hyperemic and swollen with a superficial white membrane; examination and diagnosis revealed laryngeal chemical inhalation injury. Panel **(B)** shows that the mucosa at the base of the tongue and epiglottis were congested and smooth. The mucosa of the bilateral ventricular band and laryngeal ventricle were swollen, congested, and smooth. The mucosa of both vocal cords were swollen and smooth. Laryngeal chemical inhalation injury was diagnosed. Panel **(C)** shows that the mucosa at the base of the tongue and the epiglottis were congested and smooth. The mucosa of the bilateral ventricular band and laryngeal ventricle were swollen, congested, and smooth. Laryngeal chemical inhalation injury was diagnosed. Panel **(D)** shows that the epiglottic surface, bilateral ventricular bands, laryngeal ventricles, both vocal cords, and subglottic mucosa were hyperemic and swollen with a surface pseudomembrane. The bilateral raphe were hyperemic and swollen. Laryngeal chemical inhalation injury was diagnosed.

The clinical manifestations and laboratory examination results of the patients are shown in Tables 1–3. The results of CT and laryngoscopy are shown in Figures 1, 2. The main clinical manifestations were ocular and respiratory irritation. Chest CT showed that all eight patients had varying degrees of lung injury, mainly manifesting as exudative lesions and pleural effusion. Laboratory tests revealed arterial blood gas hypoxemia, abnormal white blood cell count, and increased neutrophil ratio. The admission diagnoses were paraquat poisoning, laryngeal chemical inhalation injury, and chemical conjunctivitis. The main conventional treatment drugs were tacrolimus (3 mg, twice per day, oral administration), dexamethasone (40 mg/day, intravenous drip), piperacillin sodium for injection and tazobactam (4.5 g, 3 times per day, intravenous drip), ulinastatin (100,000 U, every 8 h, micropump injection), polyene phosphatidylcholine (20 mL/day, intravenous drip), alanyl-glutamine (20 g/day, intravenous drip), and furosemide (20 mg, twice per day, intravenous injection). In addition, tobramycin dexamethasone eye drops were given to improve the eye symptoms. The changes in symptoms at discharge were as follows: the eye symptoms of case 1 improved, the pharyngeal pain was relieved, and the dysphagia and chest tightness resolved. In case 2, the voice normalized, the throat pain was relieved, the eye pain disappeared, and the symptoms of chest tightness and wheezing resolved. In case 3, the eye symptoms, burning sensation in the pharynx, cough, and chest tightness were all relieved. In case 4, the pharyngeal symptoms, dysphagia, chest tightness, and wheezing all improved. In case 5, the dry pharyngeal itching resolved, the voice normalized, and chest tightness improved. In case 6, the foreign body sensation in the throat disappeared, the pharyngeal pain was relieved, the tears stopped, and the chest tightness resolved. Cases 7 and 8 got relief from the foreign body sensation in the throat. Case 8 was discharged from the hospital on day 3; cases 5, 6, and 7 were discharged on day 4; and cases 1, 2, 3, and 4 were discharged on day 7 following their collective admission.

**Table 1:** Main clinical manifestations and examination results of the patients.

Case	Sex	Age	Main clinical manifestation	Laryngoscopy results	Chest CT findings
1	Female	50	Dry, painful eyes; pharyngeal pain and dysphagia; chest distress.	The mucous membranes of the bilateral ventricular bands, laryngeal ventricles, and both vocal cords were hyperemic and swollen with a superficial white membrane.	The bronchial walls of both lungs were diffusely thickened. Multiple patches, large sheets, and cord-like high-density shadows were found in both lungs.
2	Female	59	Eye pain; throat pain and hoarseness; “suffocating” chest tightness.	The mucosa of the base of the tongue and epiglottis were congested and smooth. The mucosa of the bilateral ventricular band and laryngeal ventricle were swollen, congested, and smooth. The mucosa of both vocal cords was swollen and smooth.	There were patchy, high-density lesions in both lungs. Fluid density foci were found in the pleural space.
3	Female	55	Eye pain, tearing, photophobia; burning sensation in the pharynx, cough, and chest tightness.	The mucosa of the base of the tongue and epiglottis were congested and smooth. The mucosa of the bilateral ventricular band and laryngeal ventricle were swollen, congested, and smooth.	A few patchy foci of ground glass density were seen in the left lower lobe and the posterior segment of the upper lobe.
4	Female	50	Dry eyes, pain, photophobia, pharyngeal pain, dysphagia, chest tightness, wheezing, and orthopnea.	The epiglottic surface, bilateral ventricular bands, laryngeal ventricles, both vocal cords, and subglottic region mucosa were hyperemic and swollen with surface pseudomembrane. The bilateral raphes were hyperemic and swollen.	Multiple patchy ground glass density lesions were found in the middle lobe of the right lung and lower lobes of both lungs. Fluid density foci were found in the pleural space bilaterally.
5	Female	37	Dry itchy pharynx, hoarseness; chest distress.	Laryngoscopy was not performed.	Patchy and cord-like shadows were seen in both lungs.
6	Female	36	Eye pain, tears, photophobia, foreign body sensation; foreign body sensation in pharynx, cough; chest distress.	Laryngoscopy was not performed.	A few patchy and cord-like shadows in both lungs.
7	Female	41	Sensation of a foreign body in the pharynx.	Laryngoscopy was not performed.	A few patchy foci of ground glass density were seen in the lower lobes of both lungs.
8	Female	60	Sensation of a foreign body in the pharynx.	Laryngoscopy was not performed.	There were multiple nodular and cord-like high-density lesions scattered in both lungs.

CT, computed tomography.

Laryngoscopy was performed on February 7, 2023, for cases 1, 3, and 4. For case 2, it was performed on February 8, 2023.

CT examination for cases 1 and 8 was performed on January 31, 2023; for Cases 2, 3, and 7, it was performed on January 30, 2023; for Case 4, it was performed on February 1, 2023; for Cases 5 and 6, it was performed on February 4, 2023.

**Table 2:** Results of arterial blood gas analysis of the patients on admission.

Case	pH (7.35–7.45)	PaO_2_ (83–108 mmHg)	SO_2_ (94–98%)	PCO_2_ (32–48)	BE (−3 ~ 3)	Lac (1–1.8 mmol/L)
1	7.414	61.6	92.8	34.2	−2.5	2.5
2	7.433	86.8	97.3	38.8	1.14	1.7
3	7.432	56.2	91.6	37.6	0.4	2.3
4	7.425	68	95.4	36.4	−0.65	0.9
5	7.429	84.7	97.2	38.6	0.76	1.6
6	7.441	58.3	92.1	34.8	−0.49	1.2
7	7.428	81.9	97.7	40.7	1.79	1.2
8	7.424	76.5	97	36.6	−0.58	2

PaO_2,_ Partial pressure of oxygen; SO_2,_ Oxygen saturation; PCO_2,_ Partial pressure of carbon dioxide; BE, Base excess; Lac, Lactate acid.

**Table 3:** Laboratory test results of the patients at different time-points following hospital admission.

Case	Time	WBC (×10^9^/L) 3.5–9.5	NEU (%) 40–75	ALT (IU/L) 0–35	AST (IU/L) 14–36	DBIL (μmol/L) 0–5	IBIL (μmol/L) 0–19	CK (U/L) 30–135	CK-MB (ng/ml) 0.3–4	BUN (mmol/L) 2.5–6.1	Cr (μmol/L) 46–106
1	2023.2.2	16.43	93.60	18	22	0	4	62	0.7	7.8	46
	2023.2.4	19.51	85.70	22	11	2.6	4.2	34	0.7	8.8	44
	2023.2.8	23.20	82.20	37	16	2.7	6.7	36	0.8	6.1	43
2	2023.2.2	21.09	90.50	21	25	0	8	38	1	7.5	54
	2023.2.4	13.68	86.90	24	17	4	7.9	24	1	11.9	56
	2023.2.8	17.48	80.50	31	18	4.3	8.4	33	4.2	8.1	50
3	2023.2.2	14.53	94.10	19	23	3.0	4.2	21	0.8	6.2	40
	2023.2.4	15.5	90.80	19	10	3	4.2	19	0.8	10.2	49
	2023.2.8	14.36	78.00	30	15	1.9	3.3	27	1.2	7.4	48
4	2023.2.2	17.81	95.40	21	31	0	9	104	9.8	6.8	51
	2023.2.4	20.2	91.40	30	20	2.9	5.7	94	9.8	6.9	48
	2023.2.8	12.74	62.30	28	14	3.1	5.6	42	3.2	4.8	58
5	2023.2.2	16.49	94.40	20	29	0	14	40	0.9	6.2	53
	2023.2.4	18.79	87.00	243	89	4.6	6.7	56	0.9	7.7	57
6	2023.2.2	11.8	76.60	17	30	0	7	737	1.1	5.8	56
	2023.2.4	12.47	79.70	27	21	2.6	4.6	131	1.1	7	53
7	2023.2.2	11.11	88.70	15	26	0	4	33	0.6	5.2	35
	2023.2.4	13.9	84.00	10	13	2.6	2.1	29	0.6	5.9	42
8	2023.2.2	8.98	91.70	14	22	0	6	<20	/	5.8	38

WBC, white blood cells; NEU, neutrophils; ALT, alanine transaminase; AST, aspartate aminotransferase; DBIL, direct bilirubin; IBIL, indirect bilirubin; CK, creatine kinase; CK-MB, creatine kinase-MB; BUN, blood urea nitrogen; Cr, creatinine.

Cases 1 and 2 were re-examined at our hospital on February 16, 2023. No abnormalities were found on routine blood tests or liver and kidney function examinations, and there were no significant changes in their chest CT scans compared to those performed on February 8. We therefore recommended that the patients be followed-up regularly. The remaining patients were re-examined at the local hospital, and there was no significant progress compared with previous examinations.

All eight patients were followed-up at the local hospital 1 month after their discharge, and there was no significant progress compared with previous examinations.

## 3 Discussion

Paraquat is a highly toxic compound. Owing to a lack of specific antidotes (5), the clinical treatment for paraquat poisoning is mainly symptomatic. It largely consists of removing the paraquat from the body and protecting the affected organs (6). Paraquat poisoning is characterized by rapid disease progression and a high mortality rate (7); however, its mechanism of action remains unclear. Most researchers currently believe that the main mechanism of its toxicity is based on the REDOX cycle and the generation of intracellular oxidative stress (8, 9). Paraquat is corrosive and highly irritating to epithelial cells and causes cell damage through various pathways (10–12). It can seriously damage the skin, eyes, and gastrointestinal mucosa and cause severe pain and dysphagia (10).

Paraquat can damage to the lungs, kidneys, liver, muscle tissues, and central nervous system. Of these, lung injury is the most prominent (13), as lung is the specific target. Paraquat can enter the human body in a variety of ways to cause poisoning, the most common being through the digestive tract. In recent years, exposure to paraquat through other routes, such as intravenous or intramuscular injection (14–16), ocular exposure (17), and scrotal contact, has also been reported to lead to local adverse reactions and organ function impairment. Regardless of how paraquat enters the body, the lungs selectively accumulate it. Even if its concentration in the blood begins to drop, it can persist in the lungs, destroying cellular structures and causing lung damage (4). Inhalation exposure is a relatively rare route for paraquat poisoning, and the large surface area and abundant blood supply of the lungs provide an excellent way for it to be absorbed and distributed (6). Even when inhaled in small amounts, paraquat can act directly on the lungs and cause lung damage. In a report by Lv et al. (18), it was reported that exposure to even trace amounts of paraquat can cause lung damage. Therefore, paraquat poisoning through any route should be considered very serious and should be promptly treated.

A study by Zhao et al. (19) evaluated paraquat concentrations in the urine. At 10 μg/mL, no obvious liver, kidney, or myocardial function injury was observed on laboratory examinations, and no obvious abnormalities were observed on early-stage CT scans although increased lung texture and small plaques of higher density were apparent, as well as a few cases of pleural effusion. In the middle stage of poisoning, subsequent CT scans showed small speckles of higher density, ground glass lesions, shrinkage or absorption of pleural effusion, and thickening of lung textures. Late-stage lung CT scans, however, showed no abnormalities or increased lung texture. In our case, all eight patients with paraquat poisoning showed different degrees of ocular pain, photophoresis, tearing, hoarseness, chest tightness, and other clinical manifestations. Lung imaging examinations also showed varying degrees of injury. Our patients did not show the pulmonary fibrosis considered typical of paraquat poisoning, which is similar to the presentation of the patients with paraquat poisoning reported by Zhao et al. (19), who ingested small doses of the chemical. Our patients also showed varying degrees of increased lung texture and small patches of increased density, and some showed thickening of their bronchial tube walls, glass-like lesions of pleural effusion, and pulmonary fiber lesions.

Paraquat poisoning is a serious acute toxicity that must be promptly addressed. At present, paraquat has been banned for domestic sale and use in China, but certain products still traded on the market, such as diachronium, phosphine oxalate, and other herbicides, still contain certain amounts of paraquat. Therefore, active prevention and control of paraquat poisoning is still necessary.

## Data availability statement

The original contributions presented in the study are included in the article/supplementary materials, further inquiries can be directed to the corresponding authors.

## Ethics statement

Studies involving human participants were reviewed and approved by the Ethics Committee of Shandong University Qilu Hospital. All the patients provided written informed consent to participate in this study. Written informed consent was obtained from each individual for the publication of any potentially identifiable images or data included in this article.

## Author contributions

MS: Writing – review & editing, Writing – original draft. MZ: Writing – review & editing. TJ: Investigation, Writing – review & editing. GY: Formal analysis, Writing – review & editing. AG: Writing – review & editing. XZ: Writing – review & editing. LG: Data curation, Writing – review & editing. RS: Writing – review & editing. ZZ: Writing – review & editing. TZ: Writing – review & editing. XJ: Writing – review & editing. BK: Writing – review & editing.
